# Association between blood pressure classification defined by the 2017 ACC/AHA guidelines and coronary artery calcification progression in an asymptomatic adult population

**DOI:** 10.1093/ehjopen/oeab009

**Published:** 2021-08-11

**Authors:** Ki-Bum Won, Donghee Han, Su-Yeon Choi, Eun Ju Chun, Sung Hak Park, Hae-Won Han, Jidong Sung, Hae Ok Jung, Hyuk-Jae Chang

**Affiliations:** Division of Cardiology, Ulsan University Hospital, University of Ulsan College of Medicine, Ulsan, South Korea; Division of Cardiology, Severance Cardiovascular Hospital, Yonsei-Cedars-Sinai Integrative Cardiovascular Imaging Research Center, Yonsei University College of Medicine, Yonsei University Health System, 50-1 Yonsei-ro, Seodaemun-gu, Seoul 03722, South Korea; Division of Cardiology, Severance Cardiovascular Hospital, Yonsei-Cedars-Sinai Integrative Cardiovascular Imaging Research Center, Yonsei University College of Medicine, Yonsei University Health System, 50-1 Yonsei-ro, Seodaemun-gu, Seoul 03722, South Korea; Department of Imaging and Medicine, Cedars-Sinai Medical Centre, Los Angeles, CA, USA; Division of Cardiology, Healthcare System Gangnam Centre, Seoul National University Hospital, Seoul, South Korea; Division of Radiology, Seoul National University Bundang Hospital, Seongnam, South Korea; Division of Radiology, Gangnam Heartscan Clinic, Seoul, South Korea; Department of Internal Medicine, Gangnam Heartscan Clinic, Seoul, South Korea; Division of Cardiology, Heart Stroke & Vascular Institute, Samsung Medical Centre, Seoul, South Korea; Division of Cardiology, Seoul St. Mary's Hospital, College of Medicine, The Catholic University of Korea, Seoul, South Korea; Division of Cardiology, Severance Cardiovascular Hospital, Yonsei-Cedars-Sinai Integrative Cardiovascular Imaging Research Center, Yonsei University College of Medicine, Yonsei University Health System, 50-1 Yonsei-ro, Seodaemun-gu, Seoul 03722, South Korea

**Keywords:** Blood pressure, Hypertension, Coronary artery calcium score

## Abstract

**Aims:**

Coronary artery calcium score (CACS) is widely used for cardiovascular risk stratification in asymptomatic population. We assessed the association of new blood pressure (BP) classification using the 2017 American College of Cardiology/American Heart Association guidelines with coronary artery calcification (CAC) progression according to age in asymptomatic adults.

**Methods and results:**

Overall, 10 839 asymptomatic Korean adults (23.4% aged ≤45 years) who underwent at least two CACS evaluations for health check-up were enrolled. Participants were categorized by age (≤45 and >45 years) and BP [normal (<120/<80 mmHg, untreated), elevated (120–129/<80 mmHg, untreated), Stage 1 hypertension (untreated BP 130–139/80–89 mmHg) or Stage 2 hypertension (BP ≥140/≥90 mmHg or anti-hypertensive use)] groups. CAC progression was defined as a difference of ≥2.5 between the square root (√) of the baseline and follow-up CACS. During a mean 3.3-year follow-up, the incidence of CAC progression was 13.5% and 36.3% in individuals aged ≤45 and >45 years, respectively. After adjustment for age, sex, diabetes, dyslipidaemia, obesity, current smoking, and baseline CACS, hazard ratios (95% confidence interval) for CAC progression in elevated BP, Stage 1 hypertension, and Stage 2 hypertension compared to normal BP were 1.43 (0.96–2.14) (*P* = 0.077), 1.64 (1.20–2.23) (*P* = 0.002), and 2.38 (1.82–3.12) (*P* < 0.001) in the ≤45 years group and 1.11 (0.95–1.30) (*P* = 0.179), 1.17 (1.04–1.32) (*P* = 0.009), and 1.52 (1.39–1.66) (*P* < 0.001) in the >45 years group, respectively.

**Conclusion:**

Newly defined Stage 1 hypertension is independently associated with CAC progression in asymptomatic adults regardless of age.

## Graphical abstract

**Graphical Abstract:**
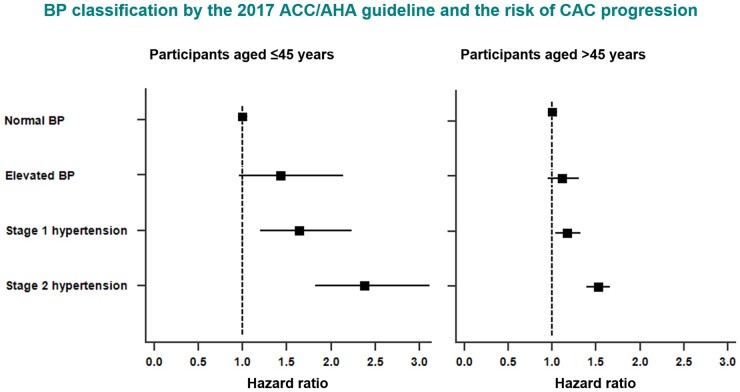

## Introduction

High blood pressure (BP) is one of the most important risk factors for cardiovascular (CV) morbidity and mortality.^1^^,^^2^ The American College of Cardiology (ACC)/American Heart Association (AHA) have recently lowered the BP thresholds for the diagnosis of hypertension; newly defining Stage 1 hypertension as a systolic BP of 130–139 mmHg or a diastolic BP of 80–89 mmHg.^3^ This reinforced criterion for hypertension could emphasize the significance of BP in preventing adverse CV events. However, the new guidelines could also lead to the overdiagnosis of hypertension and result in unnecessary treatment, particularly in young adults or adults with a low CV risk burden. Several recent studies have reported the usefulness of this new guideline,^4^^,^^5^ but there has been no consensus in clinical practice. Moreover, the additional values of reinforced BP classification for the risk of atherosclerosis progression beyond traditional risk factors remain uncertain.

In the asymptomatic population, coronary artery calcium score (CACS) has been widely used for CV risk stratification because CACS provides strong prognostic information across diverse age groups, sex, ethnicities, and baseline traditional risk factors.^6–8^ Furthermore, the progression of coronary artery calcification (CAC) is known as a significant predictor of mortality.^9^ Recent data from the Heinz Nixdorf Recall study revealed that CAC progression had an independent prognostic benefit in the absence of heavy CAC at baseline,^10^ suggesting that early detection of the presence and progression of CAC is important in reducing the risk of adverse clinical events in the asymptomatic population. However, data regarding the significance of the newly defined Stage 1 hypertension and the risk of CAC progression among different age groups are limited. Therefore, this study aimed to evaluate the association between the reinforced criteria for hypertension and CAC progression according to age in asymptomatic Korean adults.

## Methods

### Study population

In this observational, multicentre study, 10 839 self-referred asymptomatic Korean adults were enrolled from the Korea Initiatives on Coronary Artery Calcification registry. All participants had undergone at least two CAC scan examinations and had available data regarding BP, history of hypertension, and anti-hypertensive use between December 2012 and August 2016. All data were obtained during visits to the six healthcare centres. Self-reported medical questionnaires were used to retrieve information on the medical history. BP was measured using an automatic manometer on the right arm after resting for at least 5 min. Height and weight were measured with the participants wearing light clothing without shoes. Body mass index (BMI) was calculated as weight (kg)/height (m^2^). All blood samples, including total cholesterol, triglyceride, high-density lipoprotein cholesterol (HDL-C), low-density lipoprotein cholesterol (LDL-C), creatinine, glucose, and glycated haemoglobin A1C (HbA1C) were obtained after at least 8 h of fasting.

Participants were categorized by age (≤45 and >45 years) and BP [normal BP (untreated systolic/diastolic < 120/<80 mmHg), elevated BP (untreated systolic/diastolic = 120–129/<80 mmHg)], Stage 1 hypertension (untreated systolic/diastolic BP = 130–139/80–89 mmHg) or Stage 2 hypertension (systolic/diastolic BP ≥ 140/≥90 mmHg or taking anti-hypertensive medication).^3^ Diabetes was defined either as a fasting glucose level of ≥126 mg/dL, HbA1C of ≥6.5%, a previous diagnosis of diabetes, and/or taking anti-diabetic medication.^11^ Dyslipidaemia was defined as a total cholesterol level of ≥240 mg/dL, a serum LDL-C level of ≥130 mg/dL, a serum HDL-C level of ≤40 mg/dL, a serum triglyceride level of ≥150 mg/dL, and/or taking anti-hyperlipidaemic medication.^12^ Obesity was defined as a BMI of ≥25.0 kg/m^2^.^13^ Current smoking was defined as those who currently smoked or had smoked until 1 month before the study.^12^

Coronary artery calcium score was measured using the scoring system previously described by Agatston *et al.*^14^ Coronary artery calcification progression was determined using the SQRT method, defined as a difference of ≥2.5 between the square root (√) of the baseline and follow-up CACS (Δ√transformed CACS),^9^^,^^15^ considering the proportion of CACS of 0 at baseline in the present study. Annualized Δ√transformed CACS was defined as Δ√transformed CACS divided by the inter-scan period. Computed tomography (CT) to evaluate CACS was performed using scanners with a ≥16-slice multi-detector Siemens 16-slice Sensation (Siemens, Forchheim, Germany), Philips Brilliance 256 iCT (Philips Healthcare, Cleveland, OH), Philips Brilliance 40-channel multi-detector CT (Philips Healthcare), and GE 64-slice Lightspeed (GE Healthcare, Milwaukee, WI, USA). All CAC scans were performed using a scan protocol of standard ECG-triggering methods. For each centre, CACS was evaluated by the experienced CV radiologists and the results of CACS measurement were reported in the electronic health record, per routine clinical care in South Korea. Informed consent was obtained from each participant. The appropriate institutional review board committees of each centre approved the protocol of the present study.

### Statistical analysis

Continuous variables are presented as mean ± standard deviation. Categorical variables are presented as absolute values and percentage. One-way analysis of variance was used to compare continuous variables. The χ^2^ or Fisher’s exact test was used to compare categorical variables, as appropriate. Univariate linear regression analysis was performed to identify the association of clinical variables with the annualized Δ√transformed CACS. Multiple Cox regression models were used to assess the risk of CAC progression in elevated BP, Stage 1 hypertension, and Stage 2 hypertension versus normal BP; Model 1 provided the unadjusted hazard ratio (HR) and Model 2 provided the adjusted HR after considering the traditional risk factors of age, sex, diabetes, dyslipidaemia, obesity and current smoking, and baseline CACS. All statistical analyses were performed using SAS version 9.1.3 (SAS Institute Inc., Cary, NC, USA). A *P*-value of <0.05 was considered significant in all analyses.

## Results

### Baseline characteristics

The mean age of the 10 839 participants (9154 men, 84.5%) was 51.5 ± 8.6 years. The proportion of participants aged >45 years was 76.6%. The proportions of participants with normal BP, elevated BP, Stage 1 hypertension, and Stage 2 hypertension were 38.2%, 8.9%, 17.0%, and 35.9%, respectively. The overall prevalence of diabetes, dyslipidaemia, obesity, and current smoking were 14.3%, 68.1%, 41.8%, and 28.6%, respectively. Age, BMI, levels of triglyceride, glucose, and HbA1C as well as the prevalence of diabetes, dyslipidaemia, and obesity significantly increased with a higher BP classification. In contrast, the levels of HDL-C significantly decreased with a higher BP classification (*Table 1*).

**Table 1:** Baseline characteristics

	Overall (*N* = 10 839)	BP classification				
		Normal BP (*N* = 4138)	Elevated BP (*N* = 968)	Stage 1 Hypertension (*N* = 1838)	Stage 2 Hypertension (*N* = 3895)	Overall *P*-value
Age (years)	51.5 ± 8.6	49.6 ± 7.8	49.7 ± 8.8	50.2 ± 8.1	54.6 ± 8.7^*^	<0.001
Age >45 years, *n* (%)	8304 (76.6)	2947 (71.2)	643 (66.4)	1335 (72.6)	3379 (86.8)	<0.001
Male, *n* (%)	9154 (84.5)	3304 (79.8)	818 (84.5)	1639 (89.2)	3393 (87.1)	<0.001
Systolic BP (mmHg)	119.6 ± 15.0	106.9 ± 8.1^*^	123.6 ± 2.9^*^	126.8 ± 7.8	128.6 ± 15.8^*^	<0.001
Diastolic BP (mmHg)	74.9 ± 10.6	66.9 ± 6.8^*^	73.4 ± 4.4^*^	81.9 ± 4.6	80.6 ± 11.1^*^	<0.001
BMI (kg/m^2^)	24.5 ± 2.8	23.6 ± 2.5^*^	24.5 ± 2.7	24.7 ± 2.6	25.4 ± 2.8^*^	<0.001
Diabetes, *n* (%)	1546 (14.3)	372 (9.0)	93 (9.6)	195 (10.6)	886 (22.7)	<0.001
Dyslipidaemia, *n* (%)	7377 (68.1)	2558 (61.8)	638 (65.9)	1270 (69.1)	2911 (74.7)	<0.001
Obesity, *n* (%)	4526 (41.8)	1217 (29.4)	391 (40.4)	818 (44.5)	2100 (54.0)	<0.001
Current smoking, *n* (%)	3018 (28.6)	1311 (32.5)	273 (28.8)	481 (26.7)	953 (25.3)	<0.001
Total cholesterol (mg/dL)	197.4 ± 34.1	196.2 ± 33.6^*^	200.8 ± 33.2	203.1 ± 33.5	195.2 ± 34.7^*^	<0.001
Triglyceride (mg/dL)	142.1 ± 89.5	129.2 ± 78.6^*^	140.4 ± 88.0^*^	151.5 ± 104.0	151.9 ± 91.6	<0.001
HDL-C (mg/dL)	53.7 ± 16.4	54.6 ± 16.4^*^	54.0 ± 17.2	53.4 ± 15.9	52.6 ± 16.5	<0.001
LDL-C (mg/dL)	121.5 ± 31.8	121.8 ± 31.5^*^	123.3 ± 32.1	124.7 ± 31.4	119.3 ± 32.2^*^	<0.001
Glucose (mg/dL)	98.0 ± 20.5	94.0 ± 17.8^*^	96.8 ± 19.2	98.7 ± 20.7	102.2 ± 22.6^*^	<0.001
HbA1C (%)	5.7 ± 0.7	5.6 ± 0.7^*^	5.6 ± 0.7	5.6 ± 0.7	5.8 ± 0.8^*^	<0.001

Values are presented as the mean ± standard deviation or number (%).

* *P* < 0.05 vs. stage 1 hypertension in the Bonferroni’s *post hoc* test.

BMI, body mass index; BP, blood pressure; HbA1C, glycated haemoglobin A1C; HDL-C, high-density lipoprotein cholesterol; LDL-C, low-density lipoprotein cholesterol.

**Figure 1:**
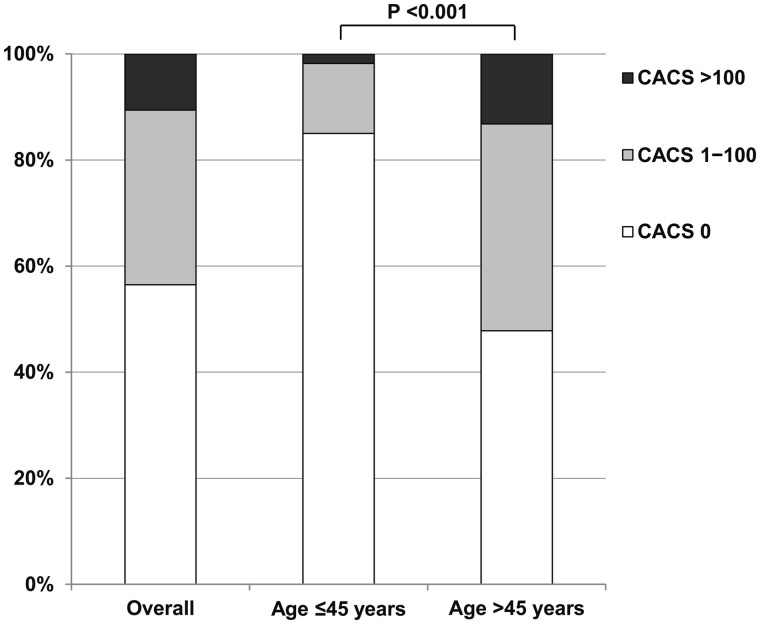
Baseline categorical coronary artery calcium score (CACS).

Among the overall participants, the mean value of baseline CACS was 45.9 ± 174.1, and the proportions of CACS of 0, 1–100, and >100 were 56.5%, 32.9%, and 10.6%, respectively. In participants aged ≤45 years, the mean value of baseline CACS was 6.9 ± 39.9, and the proportions of CACS of 0, 1–100, and >100 were 85%, 13.2%, and 1.8%, respectively. In participants aged >45 years, the mean value of baseline CACS was 57.8 ± 196.1 and the proportions of CACS of 0, 1–100, and >100 were 47.8%, 39.0%, and 13.2%, respectively (*Figure 1*).

### Changes in coronary artery calcification according to the classification of blood pressure

**Figure 2:**
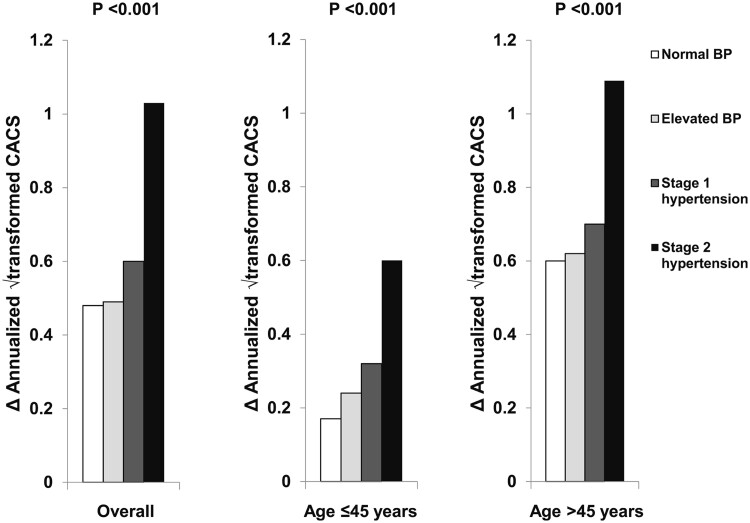
Comparison of coronary artery calcification (CAC) changes according to blood pressure (BP) classification.

The overall incidence of CAC progression in participants with normal BP, elevated BP, Stage 1 hypertension, and Stage 2 hypertension were 24.5%, 24.6%, 27.0%, and 41.4%, respectively. CAC progression was more frequently observed in participants aged >45 years than in those aged ≤45 years (36.3% vs. 13.5%; *P* < 0.001). In overall participants, the mean values of annualized Δ√transformed CACS for normal BP, elevated BP, Stage 1 hypertension, and Stage 2 hypertension were 0.48 ± 1.70, 0.49 ± 1.33, 0.60 ± 1.32, and 1.03 ± 2.14, respectively. The mean values of annualized Δ√transformed CACS were significantly different among the BP groups in participants aged ≤45 years (normal BP: 0.17 ± 0.73 vs. elevated BP: 0.24 ± 0.68 vs. Stage 1 hypertension: 0.32 ± 1.11 vs. Stage 2 hypertension: 0.60 ± 1.36; *P* < 0.001) and in those aged >45 years (normal BP: 0.60 ± 1.95 vs. elevated BP: 0.62 ± 1.55 vs. Stage 1 hypertension: 0.70 ± 1.37 vs. Stage 2 hypertension: 1.09 ± 2.23; *P* < 0.001) (*Figure 2*).

### Association between clinical variables and annualized Δ√transformed coronary artery calcium score

*Table 2* shows that age, male sex, systolic BP, BMI, current smoking, and the serum levels of triglyceride, HDL-C, glucose, and HbA1C were significantly associated with the annualized Δ√transformed CACS in participants aged ≤45 years and >45 years. Diastolic BP and the serum levels of LDL-C had significant associations with the annualized Δ√transformed CACS in participants aged ≤45 years only (*Table 2*).

**Table 2:** Association between clinical variables and the annualized Δ√transformed CACS

	Overall		Age ≤45 years		Age >45 years	
	β (95% CI)	*P*-value	β (95% CI)	*P*-value	β (95% CI)	*P*-value
Age, per-1 year increase	0.040 (0.036–0.044)	<0.001	0.031 (0.021–0.041)	<0.001	0.041 (0.035–0.047)	<0.001
Male sex	0.492 (0.398–0.586)	<0.001	0.372 (0.275–0.468)	<0.001	0.496 (0.376–0.617)	<0.001
Systolic BP	0.006 (0.004–0.008)	<0.001	0.009 (0.006–0.011)	<0.001	0.005 (0.002–0.008)	0.001
Diastolic BP	0.005 (0.002–0.009)	<0.001	0.012 (0.008–0.015)	<0.001	0.002 (−0.002 to 0.006)	0.358
BMI, per-1 kg/m^2^ increase	0.059 (0.046–0.071)	<0.001	0.047 (0.035–0.059)	<0.001	0.061 (0.045–0.077)	<0.001
Current smoking	0.102 (0.025–0.179)	0.009	0.124 (0.044–0.204)	0.002	0.160 (0.061–0.259)	0.002
Triglyceride, per-1 mg/dL increase	0.001 (0.001–0.002)	<0.001	0.002 (0.001–0.002)	<0.001	0.001 (0.001–0.002)	<0.001
HDL-C, per-1 mg/dL increase	−0.003 (−0.005 to 0.001)	0.001	−0.005 (−0.007 to −0.002)	<0.001	−0.003 (−0.006 to −0.001)	0.029
LDL-C, per-1 mg/dL increase	0.002 (0.001–0.003)	<0.001	0.003 (0.002–0.004)	<0.001	0.001 (−0.001 to 0.002)	0.167
Glucose, per-1 mg/dL increase	0.009 (0.007–0.010)	<0.001	0.007 (0.005–0.009)	<0.001	0.008 (0.006–0.010)	<0.001
HbA1C, per-1% increase	0.255 (0.204–0.306)	<0.001	0.196 (0.127–0.266)	<0.001	0.241 (0.181–0.300)	<0.001

BMI, body mass index; BP, blood pressure; CACS, coronary artery calcium score; CI, confidence interval; HbA1C, glycated haemoglobin A1C; HDL-C, high-density lipoprotein cholesterol; LDL-C, low-density lipoprotein cholesterol.

### Association between blood pressure and the risk of coronary artery calcification progression

The results of the Cox regression models for the association between BP and the risk of CAC progression are presented in *Table 3*. Model 1 showed that the risk of CAC progression was significantly increased in those with elevated BP, Stage 1 hypertension, and Stage 2 hypertension compared with those with normal BP in participants aged ≤45 years. In participants aged >45 years, the risk of CAC progression was significantly increased in Stages 1 and 2 hypertension, but not in those with an elevated BP, compared with those with normal BP levels. In model 2, Stages 1 and 2 hypertension, but not elevated BP, were significantly associated with an increased risk of CAC progression compared with those with normal BP levels after adjusting for age, sex, diabetes, dyslipidaemia, obesity, current smoking, and baseline CACS in participants aged ≤45 years and >45 years. The results of the restricted cubic spine analysis for the association between systolic and diastolic BP and the risk of CAC progression in overall participants are presented in Supplementary material online, *Figure S1*.

**Table 3:** Cox regression models for the association between BP and the risk of CAC progression

	Overall		Age ≤45 years		Age >45 years	
	HR (95% CI)	*P*-value	HR (95% CI)	*P*-value	HR (95% CI)	*P*-value
Model 1						
Normal BP	1	—	1	—	1	—
Elevated BP	1.20 (1.04–1.38)	0.011	1.58 (1.06–2.34)	0.024	1.16 (0.99–1.34)	0.062
Stage 1 hypertension	1.27 (1.14–1.42)	<0.001	1.83 (1.35–2.47)	<0.001	1.20 (1.07–1.35)	0.002
Stage 2 hypertension	2.09 (1.93–2.26)	0.001	3.12 (2.42–4.02)	<0.001	1.84 (1.69–1.99)	<0.001
Model 2						
Normal BP	1	—	1	—	1	—
Elevated BP	1.14 (0.99–1.31)	0.077	1.43 (0.96–2.14)	0.077	1.11 (0.95–1.30)	0.179
Stage 1 hypertension	1.20 (1.08–1.34)	0.001	1.64 (1.20–2.23)	0.002	1.17 (1.04–1.32)	0.009
Stage 2 hypertension	1.58 (1.46–1.72)	<0.001	2.38 (1.82–3.12)	<0.001	1.52 (1.39–1.66)	<0.001

BP, blood pressure; CAC, coronary artery calcification; CACS, coronary artery calcium score; CI, confidence interval; HR, hazard ratio.

Model 1: unadjusted.

Model 2: adjusted for age, sex, diabetes, dyslipidaemia, obesity, current smoking, and baseline CACS.

## Discussion

With the community-based cohort data from six health care centres in South Korea, Stage 1 hypertension, newly defined by the 2017 ACC/AHA guideline, was independently associated with an increased risk of CAC progression in asymptomatic adults aged ≤45 years as well as in those aged >45 years. Considering that CAC progression is a useful prognostic marker in asymptomatic participants, the recently reinforced BP classification system using the ACC/AHA guideline may help stratify CV risk beyond the traditional risk factors.

Most young adults with Stage 1 hypertension, as defined by the new (2017) ACC/AHA guideline, were considered as having a low CV risk burden and would not be recommended for pharmacological treatment. If Sage 1 hypertension is not related to the risk of adverse clinical events in young adults or adults with low CV risk burden, overdiagnosis of hypertension related to the reinforced BP criteria can result in unnecessary treatment and increased medical costs. In the recent Coronary Artery Risk Development in Young Adults study,^4^ Yano *et al.* reported that elevated BP, Stage 1 hypertension, and Stage 2 hypertension occurring before 40 years of age were associated with a significantly higher risk of CV events than normal BP (HR 1.67, 1.75, and 3.49, respectively) during a median follow-up of 18.8 years. A pooled prospective cohort analysis of 154 407 Chinese adults identified consistent results, where Stage 1 hypertension was significantly associated with an increased risk of CV mortality, particularly among young adults and those without a history of CV disease during a total follow-up of 1 718 089 person-years.^5^ Consistently, Son *et al.*^16^ reported that compared with a normal BP, Stages 1 and 2 hypertension were associated with an increased risk of subsequent CV events among 2 488 101 Korean adults aged 20–39 years. These findings imply that the recent ACC/AHA BP classification system can assist CV risk stratification.

Coronary artery calcium score has been widely used for estimating CV risk in the asymptomatic adult population due to its powerful prognostic information across diverse clinical conditions.^6–8^ In particular, the low cost, low inter- and intra-observer variability, high reproducibility across various vendors, and easily obtainable images make CACS an attractive tool for CV risk stratification.^6^^,^^17–20^ A recent cross-sectional study of 96 166 Koreans reported that the higher BP categories defined by the 2017 ACC/AHA guidelines were positively related to prevalent CAC and that correlation began in the elevated BP category, even in a young, low-risk adult population.^21^ However, data on the significance of the reinforced BP criteria regarding CAC changes are limited. Budoff *et al.*^9^ previously identified that CAC progression, particularly when defined by the SQRT method, added incremental predictive value for all-cause mortality over baseline CACS, time between scans, demographics, and CV risk factors. Recently, Lehmann *et al.*^10^ reported that CAC progression did not have a predictive value for the risk of adverse CV events in cases of CACS of >400 at baseline. In the present study, the proportion of participants with baseline CACS of >400 was only 2.6%. Despite a significant difference in the incidence of CAC progression between participants aged ≤45 years and those aged >45 years, the annual changes in CAC were found to be increased with a higher BP classification in both age groups. Moreover, stage considering the traditional risk factors and baseline CACS together in both age groups. Based on the results from the Heinz Nixdorf Recall study, which emphasized the significance of prehypertension defined by the Joint National Committee 7 guideline on accelerated CAC progression,^22^ a stricter BP control to meet the enhanced criteria for hypertension could attenuate the progression of coronary atherosclerosis. Furthermore, this therapeutic strategy may have benefits for the asymptomatic young adult population, considering that atherosclerotic CV events commonly occur in conditions with low CV risk burden.^23–26^

The necessity of pharmacological intervention increases in the middle-aged and older population because the prevalence of metabolic abnormalities gradually increases with age.^27^ Pharmacological agents could have an effect on the progression of coronary atherosclerosis;^28^^,^^29^ for this reason, coronary CT angiography (CCTA) has been proposed to have additional benefits over CACS and the traditional risk factors in asymptomatic patients due to its ability of providing more detailed coronary atherosclerotic information, including luminal stenosis severity and plaque composition. However, recent data from the Coronary CT Angiography Evaluation For Clinical Outcomes: An International Multicenter (CONFIRM) registry revealed that further prognostic benefit was not conferred by CCTA when considering the traditional risk factors and CACS in an asymptomatic population during a mean follow-up of 5.9 ± 1.2 years.^30^ Compared with the CV risk stratification based on the traditional risk factors and CACS, the role of CCTA may be limited in the asymptomatic adult population.

In contrast to the new ACC/AHA guideline, the 2018 European Society of Cardiology (ESC)/European Society of Hypertension (ESH) guidelines have retained the traditional definition of hypertension.^31^ Under the paucity of consensus regarding the definition of hypertension in recent clinical practice, several studies have suggested the use of CACS to determine individualized therapeutic goals of BP in the treatment of hypertension,^32–34^ as recent studies have shown the usefulness of CACS use in other diverse clinical situations.^35–37^ It may be possible that CACS helps to identify hypertensive patients who may benefit from intensive BP control and hence reconcile the target BP differences between the ACC/AHA and ESC/ESH guidelines. Further outcome studies are necessary to support this approach.

There are several limitations to the present study. First, all participants voluntarily visited the hospital for a general health examination. Thus, a selection bias may be present. Second, the proportion of participants aged ≤45 years was only 23.4%. However, despite the relatively small sample size of participants aged ≤45 years, the present study identified a significant association between the newly defined Stage 1 hypertension and CAC progression beyond the traditional risk factors in this population. Third, the use of pharmacological agents was not controlled during the follow-up period due to the observational design of this study. Fourth, different CT scanners were used among the participating centres; however, all participants were examined using the same CT scanner with identical ECG-triggering method during the initial and follow-up image acquisitions. In addition, CAC progression was defined using the SQRT method, considering inter-scan variability in the present study. Fifth, the current study did not perform the variability analysis based on the firm evidence regarding variability and reproducibility of CACS measurement.^6^^,^^17^^,^^19^ Finally, the generalizability of our results may be limited considering that all participants were Korean.

## Lead author biography

**Figure:**
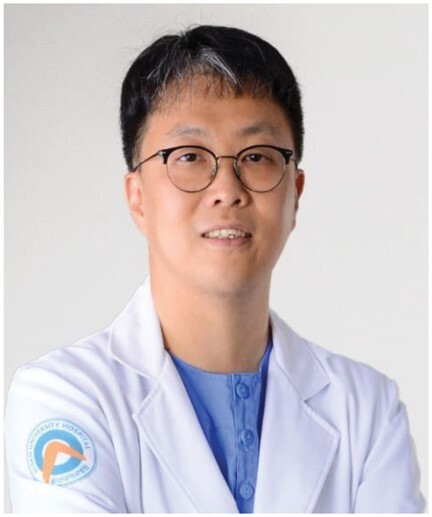


Ki-Bum Won is Assistant Professor of Cardiology at Ulsan University Hospital, University of Ulsan College of Medicine, South Korea. Dr. Won is an expert in cardiac computed tomography and coronary artery disease. He has been studying metabolic syndrome, diabetes mellitus, and atherosclerosis with non-invasive imaging tools and biomarkers focusing on the primary prevention for over ten years. Dr. Won has published about 40 research papers as first or corresponding author in the field of atherosclerotic cardiovascular disease.

## Conclusions

Stage 1 hypertension, newly defined by the 2017 ACC/AHA guideline, is independently associated with an increased risk of CAC progression in asymptomatic Korean adults, irrespective of age. The reinforced ACC/AHA guideline for the diagnosis of hypertension may assist CV risk stratification beyond the traditional risk factors in the asymptomatic adult population.

## Supplementary material

Supplementary material is available at *European Heart Journal Open* online.

## Supplementary Material

oeab009_Supplementary_Data
