# Supplementary figures and images for: Association between blood pressure classification defined by the 2017 ACC/AHA guidelines and coronary artery calcification progression in an asymptomatic adult population
Source: Eur Heart J Open. 2021 Aug 11;1(1):oeab009. doi: 10.1093/ehjopen/oeab009 (PMC9242050; doi:10.1093/ehjopen/oeab009)

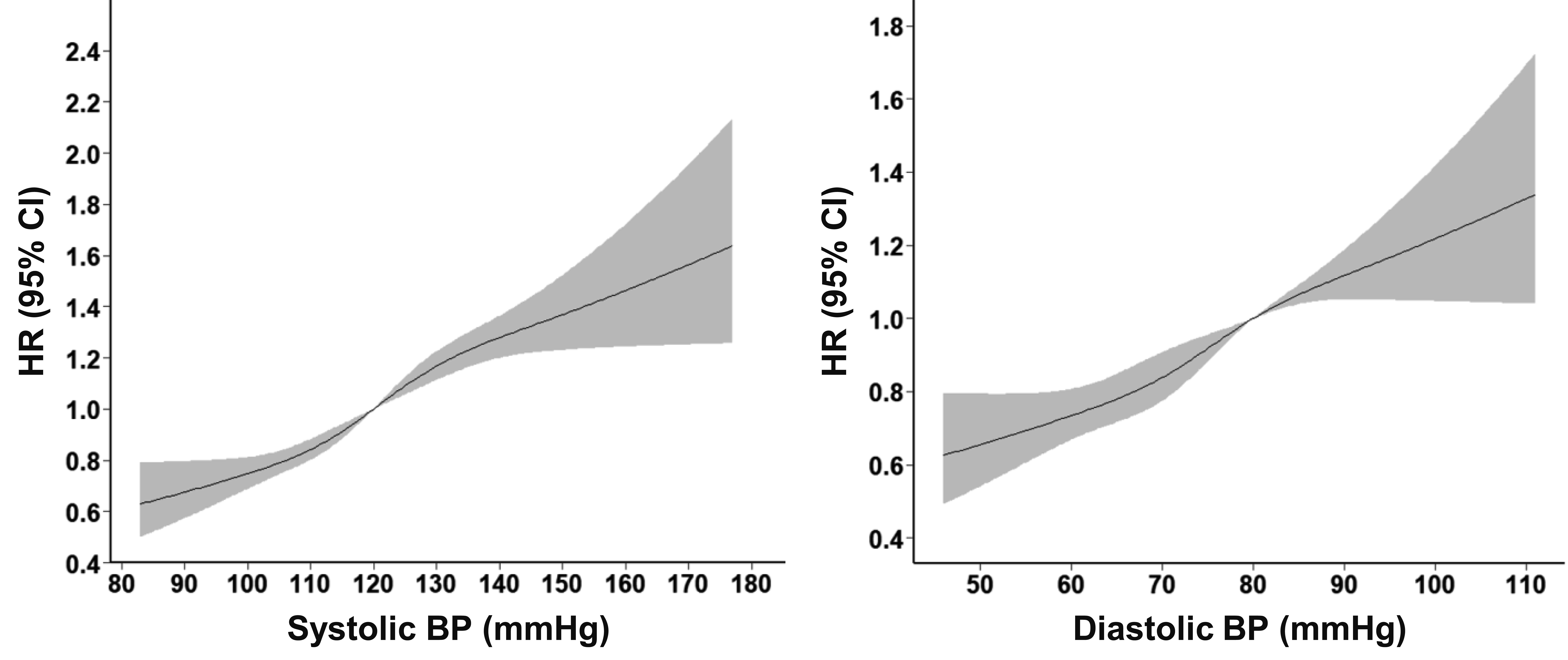

Supplement: oeab009_Supplementary_Data [file oeab009_supplementary_data.jpeg]
